# The Tryptophan Pathway Targeting Antioxidant Capacity in the Placenta

**DOI:** 10.1155/2018/1054797

**Published:** 2018-07-22

**Authors:** Kang Xu, Gang Liu, Chenxing Fu

**Affiliations:** ^1^Laboratory of Animal Nutritional Physiology and Metabolic Process, Key Laboratory of Agro-ecological Processes in Subtropical Region, Institute of Subtropical Agriculture, Chinese Academy of Sciences, National Engineering Laboratory for Pollution Control and Waste Utilization in Livestock and Poultry Production, Changsha, Hunan 410125, China; ^2^College of Animal Science and Technology, Hunan Agricultural University, Changsha, Hunan 410128, China; ^3^Hunan Collaborative Innovation Center for Utilization of Botanical Functional Ingredients and Hunan Collaborative Innovation Center of Animal Production Safety, Changsha, Hunan 410128, China

## Abstract

The placenta plays a vital role in fetal development during pregnancy. Dysfunction of the placenta can be caused by oxidative stress and can lead to abnormal fetal development. Preventing oxidative stress of the placenta is thus an important measure to ensure positive birth outcomes. Research shows that tryptophan and its metabolites can efficiently clean free radicals (including the reactive oxygen species and activated chlorine). Consequently, tryptophan and its metabolites are suggested to act as potent antioxidants in the placenta. However, the mechanism of these antioxidant properties in the placenta is still unknown. In this review, we summarize research on the antioxidant properties of tryptophan, tryptophan metabolites, and metabolic enzymes. Two predicted mechanisms of tryptophan's antioxidant properties are discussed. (1) Tryptophan could activate the phosphorylation of p62 after the activation of mTORC1; phosphorylated p62 then uncouples the interaction between Nrf2 and Keap1, and activated Nrf2 enters the nucleus to induce expressions of antioxidant proteins, thus improving cellular antioxidation. (2) 3-Hydroxyanthranilic acid, a tryptophan kynurenine pathway metabolite, changes conformation of Keap1, inducing the dissociation of Nrf2 and Keap1, activating Nrf2 to enter the nucleus and induce expressions of antioxidant proteins (such as HO-1), thereby enhancing cellular antioxidant capacity. These mechanisms may enrich the theory of how to apply tryptophan as an antioxidant during pregnancy, providing technical support for its use in regulating the pregnancy's redox status and enriching our understanding of amino acids' nutritional value.

## 1. Background

During pregnancy, the placenta's nutrient transport and barrier functions have been shown to affect the development and health of the fetus [1]. Because of its import, risks to the placenta can result in further complications. The placenta's oxidative stress leads to metabolic abnormalities, which can generate harmful effects and impede the fetal nutrition and barrier protection functions [2]. A number of studies have indicated that oxidative stress plays an important role in preeclampsia, fetal distress, fetal growth restriction, pathological abortion, and other pregnancy-associated diseases [3].

In the placenta, syncytiotrophoblast mainly serves invasion and endocrine functions but may also be an important part of the placental barrier, ensuring optimal fetal development and pregnancy. During gestation, various factors can induce oxidative stress in syncytiotrophoblast, trophoblast, and other portions of the placenta, thus disturbing the placenta's overall functioning; even worse, these can induce pathological pregnancy [4]. For instance, unreasonable nutrient intake, inflammation, heat stress, high stocking density, ultraviolet radiation, placental ischemia, and other harmful factors can induce oxidative stress on the placenta during pregnancy [5–8].

In addition to a myriad of external factors, there are numerous internal factors that can affect placental stress. The addition of antioxidants into a diet during pregnancy is important to improve antioxidant capacity of the placenta and fetus and is beneficial for the mother's health. Some traditional antioxidant nutrients (such as vitamin C and vitamin E) are used in the diet and can relieve oxidative stress during pregnancy [9]. However, these traditional antioxidants also have side effects at the population level, including reduced fetal weight, increased blood pressure in pregnant women, and increased risk of premature rupture of membranes [9–11]. The exploration and application of new antioxidative additives in the diet are thus important and significant to prevent oxidative stress and associated diseases during pregnancy [10, 11].

Tryptophan (Trp) is an essential amino acid in animals. It is also a precursor to many active molecules, such as serotonin, melatonin, kynurenic acid, NAD, and NAPD [12]. Studies have reported that Trp and some metabolites (melatonin, kynurenic acid, and xanthurenic acid) can act as effective antioxidants in organisms, removing reactive oxygen, reactive nitrogen, and active chlorine species and enhancing the organism's protection against free radical damage [13–15]. In endotoxic shock mice, Trp acted as an effective scavenger to clear free radicals and alleviated cellular damage caused by free radicals [16]. Watanabe et al. showed that L-Trp was an important antioxidant in the human placenta, as it helps inhibit the lipid peroxidation reaction under oxidative stress [17]. Additionally, Trp catabolism and related metabolic enzymes in the placenta were lower in patients with preeclampsia and eclampsia than in pregnant women without these conditions [18]. Animal experiments have shown that Trp supplements could reduce the mortality and abortion rate of pregnant mice infected by pseudorabies virus, improving both the fetal survival rate and the proportion of litters born alive [19].

These studies reveal that Trp can act as an effective antioxidant. However, the mechanism of how it works is still poorly understood. Accordingly, this review discusses the potential antioxidation mechanisms of Trp in the placenta and other extrahepatic tissues based on related research, with the intent of providing theoretical support and practical reference to improve the antioxidant capacity and reproductive performance of pregnant mammals by controlling Trp metabolism.

## 2. Antioxidant Property of Trp

In previous research, Trp has been found to be an important antioxidant in certain foods, such as eggs [20], yacon [21], and potatoes [22]. Recent studies have indicated that Trp contributes as an antioxidant in breast milk [23]. Additionally, Trp was proven to be a powerful antioxidant in *in vitro* culture tests of human glioma cells [24]. Under high-density feeding conditions, increased Trp in a diet (0.48%) may significantly improve the antioxidant capacity of ducks, which increased glutathione peroxidase (GSH-Px) and catalase (CAT) content in the pecloralis muscles and other tissues [25]. These studies have shown that Trp is an effective antioxidant in animals.

To investigate the mechanism of Trp's antioxidation, Perez-Gonzalez et al. showed that Trp cannot be oxidized by hydrogen peroxide across a variety of chemical environments based on quantum mechanical and chemical detection methods. The antioxidant property of Trp *in vivo* is not directly attributable to the radical scavenging activity of Trp molecules per se, but rather the radical scavenging activity of some Trp metabolites [26, 27] or the activation of antioxidative systems in the body after being treated by Trp. Christen et al. demonstrated that some hydroxylated metabolites of Trp (serotonin, 3-hydroxykynurenine, xanthurenic acid, and others) possessed radical scavenging activity, but Trp did not [14].

## 3. Antioxidant Property of Some Trp Metabolites

In the body, tryptophan (Trp) is catabolized through two primary pathways and generates numerous bioactive molecules in the following ways: (1) by synthesizing indole derivatives, containing serotonin and melatonin, through the biosynthesis pathway of melatonin, and (2) by producing kynurenic acid, xanthurenic acid, anthranilic acid, nicotinic acid, and others through the kynurenine pathway catalyzed by tryptophan 2,3-dioxygenase (TDO) in the liver or indoleamine 2,3-dioxygenase (IDO) in the extrahepatic tissues [14, 28].

Many studies have reported the antioxidant properties of Trp metabolites, especially melatonin. Melatonin can directly remove free radicals and enhance antioxidation capacity by enhancing the expression or activity of antioxidant enzymes [15]. Richter et al. reported that melatonin increased the expression of antioxidant enzymes in the placenta, improving placental efficiency and birth weight when mothers were malnourished [29]. Wang et al. found that melatonin treatment could significantly reduce LPS-induced oxidative and hypoxia stresses in the placenta [30]. Tamura et al. found that the placenta could synthesize a small amount of melatonin, stimulating the synthesis and secretion of melatonin by the pineal gland, thus relieving oxidative stress on the placenta [31].

In the kynurenine pathway, kynurenine [14], kynurenic acid [32], xanthurenic acid [33], 3-hydroxyanthranilic acid (HA), and other Trp metabolites were shown to have the ability to effectively eliminate free radicals *in vivo* and *in vitro*. Weiss et al. found that 3-hydroxyanthranilic acid can effectively remove reactive oxygen species and active chlorine [13].

## 4. Antioxidant Property of Trp Metabolic Enzymes

In addition to these kynurenine metabolites, the metabolic enzymes IDO and TDO in the kynurenine pathway are also considered “scavengers” of “free radicals” and important antioxidant enzymes. TDO and IDO use superoxide anion as a cofactor to catalyze the oxidation of Trp to kynurenines [34]. The efficiency of these two enzymes to clear free radicals is even higher than that of SOD [34]. Their enhanced activity was suggested to be a response or adaptation to oxidative stress in the body [34].

## 5. Trp Metabolism in the Placenta

The expression of IDO is closely associated with the formation of the placenta and is thus found in its greatest abundance in the placenta [35]. During pregnancy, as maternal Trp metabolism and utilization rates increase, plasma Trp concentration is reduced [36, 37] and the ratio of kynurenine/Trp increases throughout the pregnancy [18]. Kudo considered that these changes might be ascribed to increased IDO activity in the placenta, showing that the expression of IDO1 in the placenta increased as gestation proceeded in normal pregnancies [38].

In preeclampsia patients, the mRNA and protein expression of IDO and Trp metabolic activity decreased in the placenta. The decrease extent of Trp metabolic activity was found to be associated with the severity of the disease [39, 40]. Compared with those who were not pregnant, the ratio of plasma kynurenine/Trp was significantly increased in normal pregnant women, but showed no change in preeclampsia patients [18]. Nilsen et al. researched the antioxidant activity of IDO in the placenta and found that oxidative stress was associated with decreased IDO activity in the placenta of pregnant preeclampsia patients [41]. In addition, the mRNA expression of IDO1 was detected in cultured human placental cells *in vitro*. Expression increased after LPS stimulation [42].

## 6. Trp/mTORC1/Keap1-Nrf2-ARE Pathway

In terms of antioxidation, Trp plays an important regulatory role in restoring the body antioxidant system. In 2016, Jiang et al. found that the level of GSH and GPx in muscle tissue could be enhanced by feeding Trp to grass carp, verifying that some signal molecules in the Nrf2/ARE pathway and mTOR pathway were closely related to Trp-initiated increases in body antioxidant capacity [43].

### 6.1. Keap1-Nrf2-ARE

The nuclear factor erythroid 2-related factor 2/antioxidant response element (Nrf2/ARE) pathway is the most important endogenous antioxidant pathway in the body. It plays an important role in cellular redox homeostasis and cellular defense against oxidative stress [44–46]. In the Nrf2/ARE pathway, the transcription factor Nrf2 is a key to defense oxidative stress. At least two other essential components are required to induce protective responses and cytoprotective enzymes: (1) antioxidant response elements (AREs), cis-elements with core sequence: TGAG/CNNNGC [47], and (2) Kelch ECH association protein 1 (Keap1), a cytosolic repressor molecule that binds with Nrf2 in the cytoplasm, promoting proteasomal degradation of Nrf2 [46, 48].

At a resting state, Nrf2 and Keap1 molecules combine in the cytoplasm in a nonfree and constantly degraded inactive state. After being stimulated by signals, however, Keap1 and Nrf2 uncouple; Nrf2 transfers into the nucleus from the cytoplasm and heterodimerizes with members of the small musculoaponeurotic fibrosarcoma (Maf) family of transcription factors that bind with the antioxidant response elements (AREs), activating the expression of downstream antioxidant proteins and detoxifying enzymes [44]. Research has suggested that some antioxidant proteins have genes with the cis-element ARE in their promoter regions, such as superoxide dismutase (SOD), NADPH:quinone oxidoreductase 1 (NQO1), glutathione S-transferase A2 (GSTA2), heme oxygenase 1 (HO-1), and glutathione S-transferase (GST); these serve as target genes after Nrf2 activation [49].

In the process of Nrf2 activation, the dissociation of Nrf2-Keap1 is a key step [50]. The dissociation of Keap1-Nrf2 proceeds by two primary patterns: (1) the cysteine residues of Keap1 are modified by electrophiles, inducing a change in Keap1's conformation and a dissociation of Keap1-Nrf2 and then transferring Nrf2 into the nucleus to regulate the transcription of target genes [51], and (2) the phosphorylation of Keap1 and Nrf2 induced by protein kinase C (PKC) regulates the dissociation of Keap1-Nrf2 [50, 52].

### 6.2. Trp/mTORC1

The mTOR signaling pathway mainly comprises mTOR (mammalian target of rapamycin) and other series of protein kinases. mTOR participates in the composition of two structurally and functionally distinct multiprotein complexes: mTORC1 (mammalian target of rapamycin complex 1) and mTORC2 (mammalian target of rapamycin complex 2) [53]. mTORC1 is composed of the regulatory-associated protein of mTOR (raptor), a member of the FK506-binding protein (FKBP) family, FKBP38, and others [54]. Intracellularly and/or extracellularly, the mTORC1 signaling pathway can be regulated by growth factors, oxygen, energy levels, amino acids, and stress (such as endoplasmic reticulum stress, energy stress, oxidative stress, and genotoxic stress) and plays important roles in controlling numerous important cellular processes, including protein translation, lipid synthesis, stress response, and autophagy [55].

The mTORC1/S6 kinase (S6K) pathway can be activated by amino acids' entry into cells via various signaling cascades. Numerous studies have suggested that a Trp sufficiency signal was associated with activated mTOR activity in mTORC1 [56, 57]. In porcine intestinal epithelial cells, Wang et al. found that L-Trp was not catabolized but can nonetheless induce mTOR activation and increase the expression of the L-Trp transporters (solute carrier family 3 member 1 (SLC3A1), solute carrier family 6 member 14 (SLC6A14), and solute carrier family 6 member 19 (SLC6A19)) [56]. The mesenchymal stromal cells could disrupt mTOR activation by inducing the expression of indoleamine 2,3-dioxygenase (IDO) and Trp depletion, which interferes with a Trp sufficiency signal promoting cellular mTOR activation [57].

### 6.3. mTORC1/Keap1-Nrf2-ARE

The signaling adaptor p62 is an integral component in the central gated channel of the nuclear pore protein complex. It is also a key factor in mediating cellular functions due to its ability to establish interactions with multiple signaling molecules [58, 59]. The protein p62 is an integral part of the mTORC1 complex and is essential for mTORC1 activation in response to amino acids. Studies have shown that in an amino acid-dependent manner, p62 interacted with mTOR and raptor in mTORC1 and mediated amino acid signaling for the activation of S6K1 and 4EBP1 [58]. In addition, p62 was essential for mTORC1 activation in response to amino acids, but may not be essential for mTORC1 activation in response to other stimulations (such as insulin-induced stimulations) [58]. In another report, direct phosphorylation of p62 was at S351 cysteine residue by the mTOR kinase via an *in vitro* kinase assay [60]. This suggests that the increased phosphorylation of p62 might directly reflect the steady-state level of mTORC1 kinase activity when p62 was higher expressing [60]. In mouse embryonic fibroblasts (MEFs), rapamycin (a specific inhibitor of the mTOR kinase) treatment could suppress both the phosphorylation of p62 and the downregulation of Keap1 [60], while also significantly inhibiting the expression of the Nrf2 target HO-1.

Meanwhile, p62 mediates the regulation of the Keap1-Nrf2-ARE signaling pathway via kinase phosphorylation and the uncoupling of the protein kinase [61]. In mouse embryonic fibroblasts, p62 enhances Nrf2 activation by impairing Keap1 activity [61]. PF-4708671, a specific inhibitor of S6K1, induces autophagic Keap1 degradation-mediated Nrf2 activation in a p62-dependent manner, indicating that p62-dependent Nrf2 activation might play a crucial role in protecting cells from PF-4708671-mediated apoptosis [61].

p62 interacts with the Nrf2-binding site of Keap1, competitively inhibiting the interaction between Keap1 and Nrf2 and then activating the expression of numerous genes to encode antioxidant proteins and anti-inflammatory enzymes [62, 63]. Meanwhile, when p62 activates Nrf2, Nrf2 can also positively upregulate the expression of p62, implying a positive feedback loop [64].

Numerous studies have shown that the activation of mTORC1 can lead to the activation of the Keap-Nrf2-ARE pathway in a p62-dependent manner [65, 66]. Ichimura et al. showed that in the mTORC1-dependent manner, the site S351 cysteine residue in p62 was phosphorylated, leading to increases in affinity between p62 and Keap1 and inducing the separation of Keap1 and Nrf2. Then, stable Nrf2 enters the nucleus and induces the expression of the cytoprotective genes [60].

**Figure 1:**
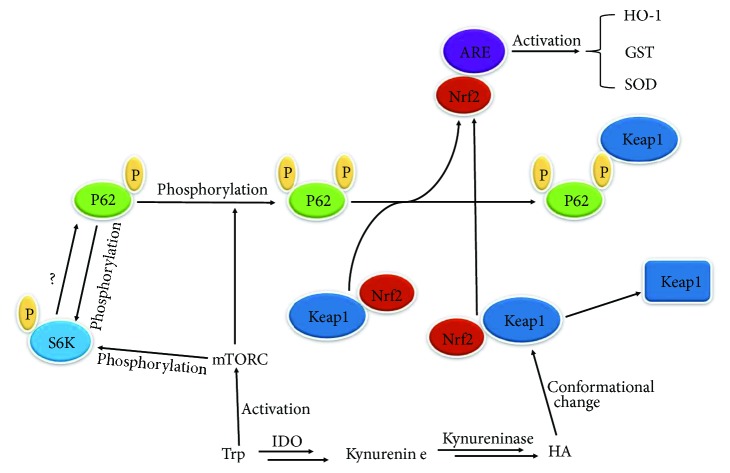
The predicted pathways where Trp exerts antioxidant effect in cell. Trp: tryptophan; IDO: indoleamine 2,3-dioxygenase; HA: 3-hydroxyanthranilic acid; HO-1: heme oxygenase 1 gene; GST: glutathione S-transferase gene; SOD: superoxide dismutase.

Accordingly, combined with the aforementioned details that Trp could enhance the expression of numerous signal molecules in the Keap-Nrf2-ARE and mTOR pathways, we suggest that in extrahepatic tissue, Trp could activate the phosphorylation of p62 after activation of mTORC1. Phosphorylated p62 would then uncouple the interaction between Nrf2 and Keap1, with activated Nrf2 then entering the nucleus to induce expressions of antioxidant proteins, thus improving the cell's antioxidation capacity (Figure 1).

## 7. The 3-Hydroxyanthranilic Acid/Nrf2-Keap Pathway

Studies have indicated that the placenta was the most enriched site for indoleamine 2,3-dioxygenase (IDO) in the kynurenine pathway and that the expression of IDO is closely related to placental formation [35]. Kudo demonstrated that IDO1 expressions in the normal placenta increased along with gestational time [38]. Other studies showed that IDO mRNA and protein expression decreases with decreased Trp metabolism in the placentas of pregnant preeclampsia patients [40]. In cultured human placenta cells, the expression of IDO1 mRNA was detected and expression increased after LPS stimulation [42]. Research has also inferred that the induction of IDO may stimulate tissues' antioxidant defense mechanisms. Transcriptome analyses of placentas from patients with preeclampsia and normal pregnancy showed that numerous expression genes were enriched in the Trp metabolism and Nrf2-Keap pathways, indicating that Trp metabolism and the Nrf2 pathway were associated with oxidative stress in the placentas of patients with preeclampsia [67]. Therefore, variations in placental Trp metabolism may affect antioxidant activity in the placenta.

**Figure 2:**
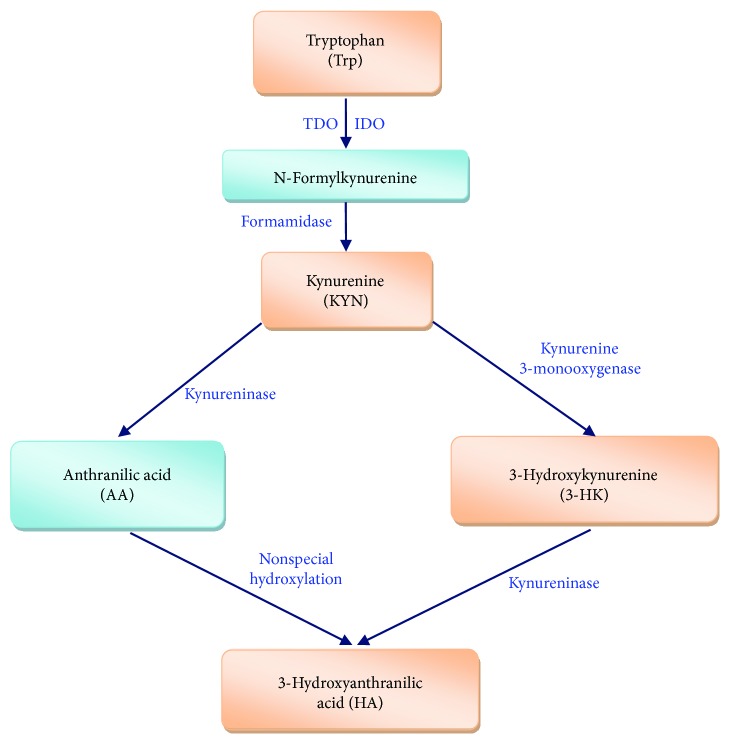
The intracellular generation pathway of 3-hydroxyanthranilic acid from tryptophan. TDO: tryptophan 2,3-dioxygenase; IDO: indoleamine 2,3-dioxygenase [19].

In the extrahepatic tissue, 3-hydroxyanthranilic acid (HA), one of Trp metabolites in the kynurenine pathway, is an effective antioxidant [68] (Figure 2). HA not only directly scavenges for free radicals (hydroxyl radical, peroxynitrite, and other radicals included) within certain density ranges but also induces the expression of heme oxygenase 1 (HO-1), a cellular protection and anti-inflammatory cytokine [68–70]. The capacity of HA to induce HO-1 expression has been shown to correlate with the formation of radicals induced by HA because the radical ROS is essential for the activation of Nrf2. The expression of the HO-1 at transcriptional level is controlled by numerous factors—for instance, the HO-1 promoter coordinates with several transcription-regulating elements in response to redox sensitivity, including Nrf2-activation. In cultured human astrocytes, HA was able to effectively induce the expression of HO-1 [70]. In human microglia, HA can weakly induce HO-1 expression and LPS-suppressed microglial HO-1 expression [70]. In mammals, inducible nitric oxide synthase (iNOS) is an isoform of NOS, which catalyzes the biosynthesis of nitric oxide (NO) [71, 72]. The production of NO involves increased ROS and reactive nitrogen species (RNS), which can induce oxidative stress, cellular damage, and inflammation [72–74]. In murine macrophages, iNOS, HO-1, and IDO were simultaneously expressed after being stimulated by interferon- (IFN-) *γ* and LPS [69]. HA dose-dependently suppressed iNOS expression by enhancing HO-1 expression and then increased IDO expression and activity [69]. In the human umbilical vein endothelial cells, HA could induce the expression of HO-1 as an antioxidant by activating the Nrf2/ARE signal pathway and suppressing the activation of NF-*κ*B, which affects the development of diseases such as vascular injury and aortic atherosclerotic inflammation [68].

Therefore, besides Trp activates the expressions of antioxidant proteins through activating the mTORC1 and Nrf2/ARE pathway, we also predict that HA could induce the expression of antioxidant proteins (such as HO-1) to enhance the cellular antioxidant capacity in the extrahepatic tissues. HA, a Trp metabolite formed along the kynurenine pathway catalyzed by IDO and kynureninase, could modify and change conformation of Keap1, induce the dissociation between Nrf2 and Keap1 after which activated Nrf2 enters into the nucleus, and induce the expression of antioxidant proteins (Figure 1). These two regulation modes may coexist in the tissues or cells and operate in coordination.

## 8. Conclusion

Overall, although Trp is suggested to be an effective antioxidant and has been used as a functional food additive, the antioxidant efficiency and antioxidant mechanism of Trp in the organism required clarification. Meanwhile, we still lack understanding of the effects of Trp, its metabolites, and metabolic enzymes on the antioxidant properties of extrahepatic tissues. This review summarized early experiments associated with the effect of Trp's antioxidant properties (also including metabolites and metabolic enzymes) to speculate on the antioxidation mechanism of Trp in the placenta. These predicted pathways pave the way for further research on Trp's antioxidant ability in the placenta and are beneficial for further exploring its antioxidation mechanism. Future findings will be useful for recommendations for Trp additives in food as an antioxidant amino acid. The present review should provide theoretical support for practical applications: to relieve oxidative stress and thus improve the reproductive outcomes for female mammals.
